# The Expression Regulatory Network in the Lung Tissue of Tibetan Pigs Provides Insight Into Hypoxia-Sensitive Pathways in High-Altitude Hypoxia

**DOI:** 10.3389/fgene.2021.691592

**Published:** 2021-10-07

**Authors:** Yanan Yang, Haonan Yuan, Tianliang Yang, Yongqing Li, Caixia Gao, Ting Jiao, Yuan Cai, Shengguo Zhao

**Affiliations:** ^1^College of Animal Science and Technology, Gansu Agricultural University, Lanzhou, China; ^2^Research on Quality Standard of Animal Husbandry, Xinjiang Academy of Animal Sciences, Xinjiang, China; ^3^State Key Laboratory of Veterinary Biotechnology, Harbin Veterinary Research Institute, Chinese Academy of Agricultural Sciences, Harbin, China; ^4^College of Grassland Science, Gansu Agricultural University, Lanzhou, China

**Keywords:** hypoxia, Tibetan pigs, PI3K-Akt pathway, MiRNA-mRNA network, lung tissue

## Abstract

To adapt to a low-oxygen environment, Tibetan pigs have developed a series of unique characteristics and can transport oxygen more effectively; however, the regulation of the associated processes in high-altitude animals remains elusive. We performed mRNA-seq and miRNA-seq, and we constructed coexpression regulatory networks of the lung tissues of Tibetan and Landrace pigs. *HBB, AGT, COL1A*2, and *EPHX*1 were identified as major regulators of hypoxia-induced genes that regulate blood pressure and circulation, and they were enriched in pathways related to signal transduction and angiogenesis, such as *HIF*-1, *PI*3*K*-*Akt, mTOR*, and *AMPK*. *HBB* may promote the combination of hemoglobin and oxygen as well as angiogenesis for high-altitude adaptation in Tibetan pigs. The expression of *MMP*2 showed a similar tendency of alveolar septum thickness among the four groups. These results indicated that *MMP*2 activity may lead to widening of the alveolar wall and septum, alveolar structure damage, and collapse of alveolar space with remarkable fibrosis. These findings provide a perspective on hypoxia-adaptive genes in the lungs in addition to insights into potential candidate genes in Tibetan pigs for further research in the field of high-altitude adaptation.

## Introduction

Tibetans are a unique and geographically isolated pig breed that inhabits the Qinghai-Tibet Plateau, which has an extreme environment with high altitudes (Wang et al., 2018; Ma et al., 2019). This unique ecological condition is characterized by low air pressure, reduced oxygen content, and high ultraviolet radiation, imposing extreme physiological challenges on domestic animals, and failure to adapt will lead to altitude illness or even death (Cao et al., 2017; Lancuo et al., 2019; Qi et al., 2019). Native high-altitude species have been selected through evolutionary processes to evolve adaptive mechanisms to cope with this harsh environment (Liu et al., 2019). Special lung properties of the Tibetan pig, yak, and Tibetan sheep living in the plateau, such as larger lungs, thicker alveolar septa, and more developed capillaries, have been previously reported by Qi and Yang (Yang et al., 2014; Qi et al., 2019). Tibetan pigs exhibit heritable adaptations to high-altitude environments as a result of natural selection. Exposure to hypoxia changes the gene profiles in various cell types and is associated with adaptation to high altitudes (Zhang T. et al., 2019). mRNAs and miRNAs are involved in many biological processes in animals, and not surprisingly, transcriptional analyses have revealed the differential expression of hypoxia regulators that enable adaptation to a hypoxic environment (Ni and Leng, 2016). The hypoxia-inducible factor-1 (HIF-1), vascular endothelial growth factor (VEGF), and mitogen-activated protein kinase (MAPK) signaling pathways are typical hypoxia-associated pathways (Lee et al., 2016; Zhang et al., 2018; Nicolas et al., 2019), and some mRNAs (*PHD*2, *VHL*, and *FIH*-1) and miRNAs (miR-363, miR-421, and miR-204) have been implicated in the regulation of the HIF-1 signaling pathway (Semenza, 2007; Ge et al., 2016; Wang et al., 2016; Xie et al., 2016).

Studies of the molecular mechanisms of livestock adaptation to high altitude have focused on miRNA-mRNA interaction networks. Here, we performed an integrative analysis of the miRNA-mRNA expression profiles in the lungs of high- and low-altitude pigs (Tibetan pigs and Landrace pigs, respectively) to identify molecular pathways and networks involved in the genetic adaptation of Tibetan pigs to hypoxic conditions.

## Materials and Methods

### Ethics Statement

All animal experiments were conducted according to the guidelines for the care and use of experimental animals established by the Ministry of Science and Technology of the People's Republic of China (Approval number: 2006–398). The procedures for animal care were approved by the Gansu Agricultural University Animal Care and Use Committee of Gansu Agricultural University, and all experiments were conducted in accordance with approved relevant guidelines and regulations.

### Sample Collection

In total, 18 Tibetan male piglets from the highlands (TH group; Gannan Tibetan Autonomous Prefecture, Gansu, representing an altitude of 3,000 m) and 18 Landrace male piglets from the lowlands (LL group; Jingchuan, Gansu, representing an altitude of 1,000 m) with similar weights and non-genetic relationships were selected, and nine piglets from each group migrated to low altitude (TL group; Tibetan pigs at low altitude) or high altitude (LH group; Landrace pigs at high altitude) from their original rearing facility at the age of 1 month. We randomly selected six pigs from each group to collect the left lower lobes of the lung from indigenous and imported adult male pigs at the age of 6 months. These animals (*n* = 6 in each group) were feed restricted for 12 h and slaughtered in their feeding place. Six samples from each group were immediately stored in stationary liquid for hematoxylin and eosin (H&E) staining, and three of the six samples were randomly selected and collected within 1 h after the pigs were harvested and stored immediately in liquid nitrogen for subsequent RNA extraction.

### Hematoxylin and Eosin Staining

Sections from the left lower lobes of the lung were stained with H&E (Ban et al., 2018; Zhang et al., 2019b), observed under a microscope (Sunny Optical Technology Co. Ltd, Ningbo, China), and then photographed using Image View (Sunny Optical Technology Co. Ltd).

### RNA Extraction

Total RNA from the lungs was extracted using a TRIzol reagent kit (Invitrogen, Carlsbad, CA, USA) according to the manufacturer's protocol, and eukaryotic mRNA was enriched by oligo (dT) beads (Epicenter, Madison, WI, USA). RNA quality was assessed on an Agilent 2100 Bioanalyzer (Agilent Technologies, Palo Alto, CA, USA) and verified by 1% gel electrophoresis. All samples presented an RNA integrity number (RIN) > 7.5.

### Library Construction and Sequencing for mRNA

After total RNA was extracted, eukaryotic mRNA was enriched by oligo (dT) beads (Epicenter) and reverse-transcribed into cDNA using random primers. mRNA was ligated with proper 5′ and 3′ adapters. The ligation products were reverse-transcribed by PCR amplification to generate a cDNA library, which was sequenced using an Illumina HiSeq™ 2500 by Gene Denovo Biotechnology Co. (Guangzhou, China).

### Library Construction and miRNA Sequencing

After total RNA was extracted for miRNA sequencing, 18–30 nt RNA molecules were enriched by polyacrylamide gel electrophoresis (PAGE). A 3′ adapter was added to enrich the 36–44 nt RNAs, and the 5′ adapter was then connected to the RNA. PCR products of 140–160 bp were amplified by reverse transcription. A cDNA library was generated and sequenced using Illumina HiSeq™ 2500 sequencing (Illumina Inc., San Diego, CA, USA) by Gene Denovo Biotechnology Co., Ltd.

### Expression Analysis of mRNAs

High-quality clean raw data were screened by removing low-quality data with fastp (Chen et al., 2018). The short-read alignment tool, Bowtie 2 (Langmead and Salzberg, 2012) was used to map reads to the ribosome RNA (rRNA) database. An index of the reference genome was built, and paired-end clean reads were mapped to *Sus scrofa* RefSeq (*Sus scrofa* 11.1) using HISAT 2 (Kim et al., 2015). The mapped reads of each sample were assembled using StringTie v1.3.1 (Pertea et al., 2015, 2016) in a reference-based approach. For each transcription region, a fragment per kilobase of transcript per million mapped reads (FPKM) value was calculated to quantify its expression abundance and variations using RSEM software. RNA differential expression analysis was performed with DESeq 2 (Love et al., 2014) software between the two groups. The raw mRNA-seq data (accession number PRJNA687172) were submitted to the Sequence Read Archive (SRA) database of NCBI.

### Expression Analysis of miRNAs

Clean reads were obtained by filtering raw reads, and all of them were aligned with small RNAs in the GenBank database (Benson et al., 2013). All the clean reads were aligned with small RNAs in the Rfam database (Griffiths-Jones et al., 2003) to identify and remove rRNAs, scRNAs, snoRNAs, snRNAs, and tRNAs. All the clean reads were also aligned with the reference genome and were searched against the miRbase database (Griffiths-Jones et al., 2006) to identify known (*Sus scrofa*) miRNAs. All the unannotated reads were aligned with the reference genome by HISAT2. 2.4. Novel miRNA candidates were identified according to their genome positions and hairpin structures predicted by mirdeep2 software. The miRNA expression levels were calculated and normalized to transcripts per million (TPM). The raw miRNA-seq data (accession number PRJNA687649) were submitted to the NCBI Sequence Read Archive (SRA) database.

### Functional Annotation of DEmRNAs

DEmRNAs were analyzed using Kyoto Encyclopedia of Genes and Genomes (KEGG) and Gene Ontology (GO) analyses using the online tool Database for Annotation, Visualization and Integrated Discovery (DAVID) (Huang et al., 2009) to explore their roles, functions, and enrichment in different biological pathways. Gene Ontology (GO) terms and pathways with q <0.05 were considered significantly enriched by DEmRNAs. The hypoxic DEmRNAs were filtered based on the intersection of our results and published hypoxia-related genes in the HIF-1 signaling pathway. The hypoxia-related genes and target genes of miRNAs were also mapped to GO terms in the GO database and pathways in the KEGG (Kyoto Encyclopedia of Genes and Genomes) database to further elucidate their functions.

### Target Prediction and Integrative Analysis of the Hypoxia-Related miRNA–mRNA Regulatory Network

We identified mRNAs with a fold change ≥2 and a false discovery rate (FDR) <0.05 as DEmRNAs. To explore more DEmiRNAs, we identified miRNAs with fold change ≥ 2 and *p* < 0.05 as DEmiRNAs. The potential target genes of DEmiRNAs were predicted using RNAhybrid 89 (version 2.1.2) + svm_light (version 6.01), miRanda (version 3.3a), and TargetScan (version 7.0), and the genes at the intersection of the results from the three software packages were selected as predicted miRNA target genes. Because mRNAs and miRNAs have potential negative regulatory relationships, we assessed the expression correlation between a miRNA and its predicted target by the Pearson correlation coefficient (PCC). Subsequently, the negatively coexpressed miRNA–mRNA pairs with PCC < −0.7 and *p* < 0.05 were screened to construct miRNA–mRNA networks.

The coexpression network diagram of DEmRNAs and DEmiRNAs was generated using the PCC, and only the relationship pair network diagram of the top 300 is shown. The coexpression network diagram of the 273 hypoxic DEmRNAs is displayed, and the correlation between miRNA and mRNA was required to account for the top 5% of the total correlation. The potential regulatory network was constructed by Cytoscape (Szklarczyk et al., 2015).

### Quantitative Real-Time PCR Validation

Total RNA from pulmonary tissues was extracted with a TRIzol reagent kit and reverse-transcribed into cDNA using a FastQuant cDNA first-strand synthesis kit (TianGen, China). SYBR® Premix Ex Taq™ II (TaKaRa, China) was used for real-time fluorescence quantitative analysis. In total, eight DEmRNAs and eight DEmiRNAs were randomly selected to determine sequencing accuracy. The primers used here were designed using Primer 5.0 software and are listed in Supplementary Tables 1, 2 (Supplementary Material 1).

The experimental data were analyzed with the 2^−Δ*ΔCT*^ method (Livak and Schmittgen, 2001). Statistical analyses were performed using GraphPad Prism 8.0 (GraphPad Software, San Diego, CA, USA) and SPSS 20.0 (SPSS, Chicago, IL, USA). The comparisons were conducted by one-way analysis of variance (ANOVA), and *p* < 0.05 was considered statistically significant.

## Results

### Morphological Structure

**Figure 1:**
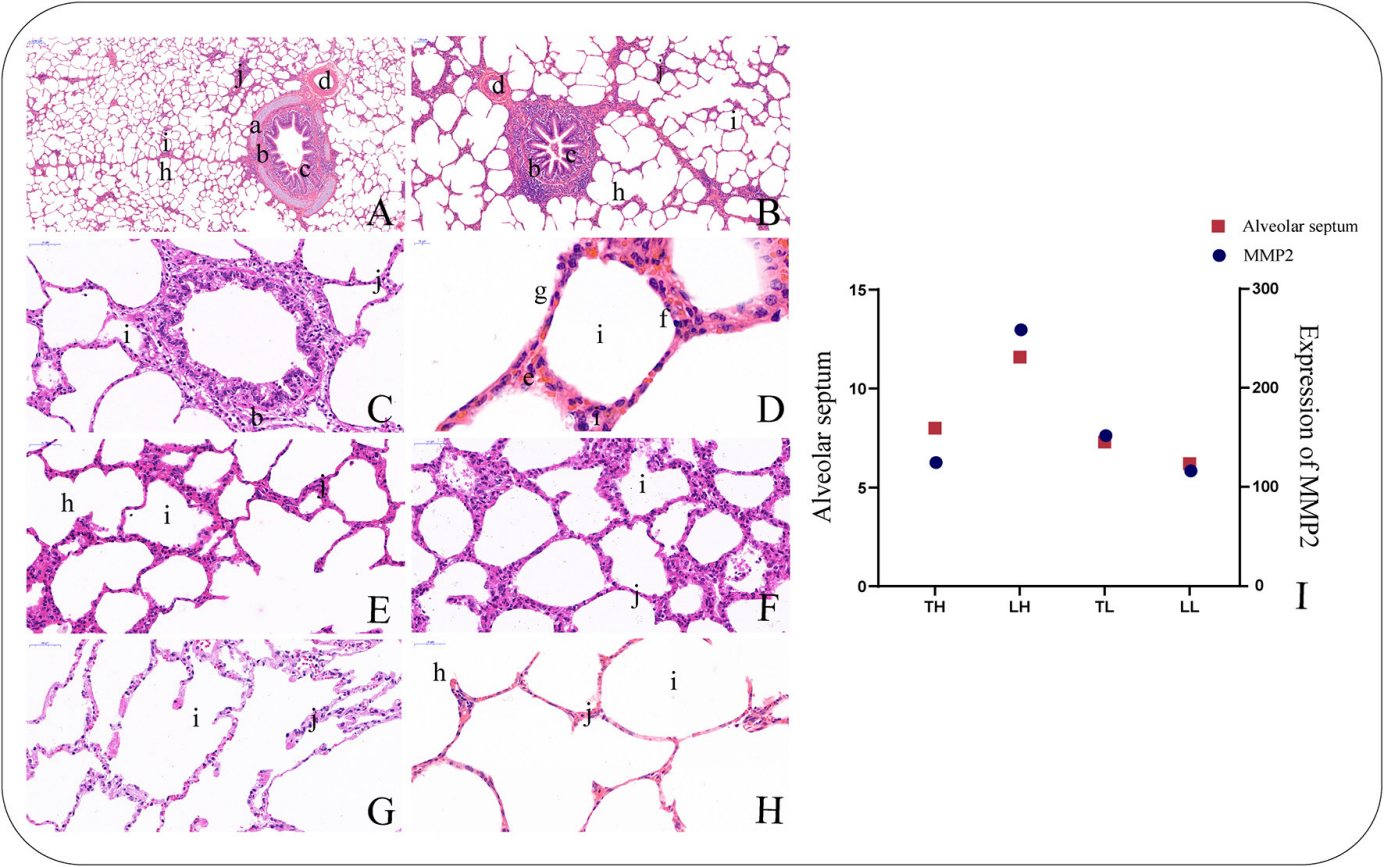
Morphological characteristics of the lungs in Tibetan pigs by H and E staining. (40×). **(A)** Morphological characteristics of small bronchiole. **(B)** Morphological characteristics of bronchiole. **(C)** Morphological characteristics of terminal bronchiole. **(D)** Morphological characteristics of alveolar cells. **(E)** Morphological characteristics of alveolar septa in the Tibetan pigs raised in highland (TH) group. **(F)** Morphological characteristics of alveolar septa in the Landrace pigs raised in highland (LH) group. **(G)** Morphological characteristics of alveolar septa in the Tibetan pigs raised in lowland (TL) group. **(H)** Morphological characteristics of alveolar septa in the Landrace pigs raised in lowland (LL) group. **(I)** The histogram shows the expression levels of *MMP*2 in the lungs of the four types of pigs and the relationship with alveolar septa. **a**. Piece of cartilage. **b**. Smooth muscle. **c**. Plica. **d**. Arteriole. **f**. Alveolar epithelial type II cells. **g**. Alveolar epithelial type I cells. **h**. Alveolar duct. **i**. Pulmonary alveoli. **j**. Alveolar septa.

H&E staining showed that the lung sections exhibited the following connective tissues with epithelia: pulmonary alveoli, smooth muscle, blood capillaries, bronchial tubes, and alveolar septa (Figures 1A–H). The sections from the TH group were characterized by smooth muscle hyperplasia and larger alveoli, while those from the LH group were characterized by a thicker alveolar septum. In addition, the analysis showed that *MMP*2 expression had a similar tendency to the alveolar septum thickness among the four groups (Figure 1I).

### Identification of DEmRNAs in the Lung

**Figure 2:**
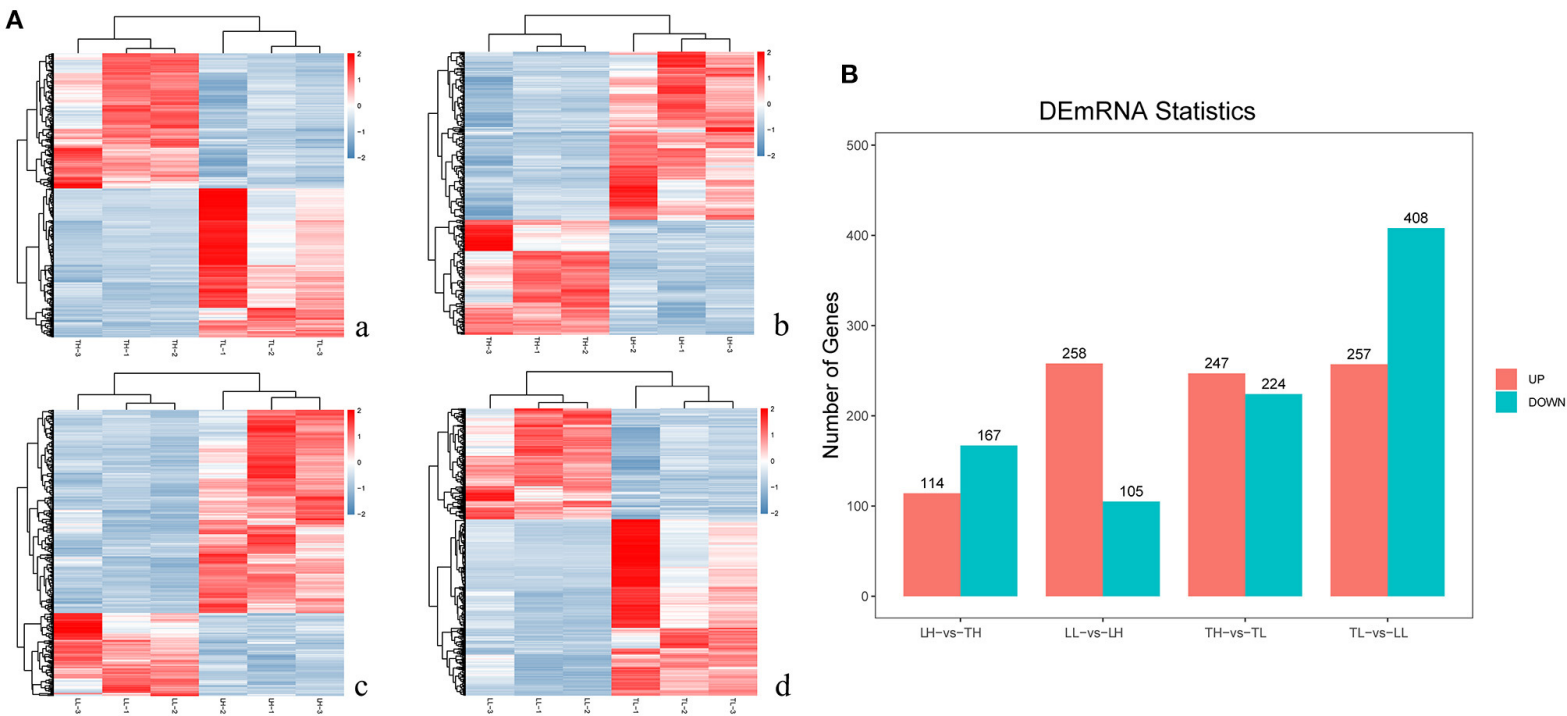
mRNA expression results among the four groups by RNA-seq. **(A)** The heatmap shows the relative expression patterns of DEmRNAs among the four groups. Each column represents a sample, and each row represents the expression levels of a single mRNA in various samples. The color scale of the heat map ranges from blue (low expression) to red (high expression). **a**. Heatmap of mRNA for Tibetan pigs raised in highland (TH) and Tibetan pigs raised in lowlands (TL). **b**. Heatmap of mRNA for TH and Landrace pigs raised in highlands (LH). **c**. Heatmap of mRNA for LH and Landrace pigs raised in lowlands (LL). **d**. Heatmap of mRNA for TL and LL. **(B)** The histogram shows the number of DEmRNAs identified among the four groups.

In total, 12 cDNA libraries, which included six Tibetan pigs and six Landrace pigs at high and low altitudes, were sequenced from lung tissues (Supplementary Material 2). After quality filtering, 51,193,662–69,112,222 clean paired reads were obtained with 99.70–99.79% of clean reads mapped to the porcine reference genome (Table 1). A total of 471 DEmRNAs (247 up- and 224 downregulated) were identified in the TH group compared to the TL group (Figure 2, Supplementary Table 3 in Supplementary Materials 1, 3). Furthermore, 809 novel genes were identified in the sequencing data. Eight mRNAs were randomly selected and detected using qRT-PCR to validate the accuracy of the sequencing data. Our verification test indicated that the qRT-PCR results were consistent with the mRNA-seq data (**Figure 4A**).

**Table 1:** Overview of the reads and quality control of the 12 libraries of the mRNA sequencing from swine lung tissue.

**Sample**	**Raw data**	**Clean data(bp)**	**Clean reads**	**Q20 (%)**	**Q30 (%)**	**GC (%)**	**Total mapped (%)**	**Unique mapped (%)**
LL-1	62,519,034	9,305,579,147	62,348,446	9,060,896,883 (97.37%)	8,641,334,794 (92.86%)	5,271,030,180 (56.64%)	59,539,505 (95.92%)	56,671,208 (91.30%)
LL-2	65,884,878	9,804,022,124	65,723,768	9,553,412,100 (97.44%)	9,118,107,584 (93.00%)	5,492,767,034 (56.03%)	61,941,659 (95.83%)	58,584,049 (90.63%)
LL-3	62,450,712	9,299,635,729	62,295,524	9,062,302,058 (97.45%)	8,648,230,615 (93.00%)	5,167,314,374 (55.56%)	58,667,319 (95.66%)	56,224,782 (91.68%)
LH-1	62,323,778	9,266,150,211	62,180,224	9,048,646,333 (97.65%)	8,658,401,070 (93.44%)	5,409,933,897 (58.38%)	59,678,317 (96.35%)	57,179,752 (92.31%)
LH-2	69,319,172	10,309,201,883	69,112,222	10,032,974,281 (97.32%)	9,562,740,889 (92.76%)	5,793,441,899 (56.20%)	65,082,781 (95.76%)	62,361,180 (91.76%)
LH-3	60,877,578	9,057,244,483	60,694,770	8,807,812,613 (97.25%)	8,387,856,985 (92.61%)	5,060,253,346 (55.87%)	56,200,627 (95.66%)	53,771,349 (91.53%)
TH-1	68,421,860	10,180,216,302	68,240,484	9,907,699,993 (97.32%)	9,443,251,897 (92.76%)	5,793,799,001 (56.91%)	64,529,051 (95.18%)	61,876,279 (91.27%)
TH-2	68,109,198	10,130,609,161	67,917,554	9,862,307,770 (97.35%)	9,401,034,430 (92.80%)	5,643,159,581 (55.70%)	64,353,888 (95.25%)	61,919,184 (91.65%)
TH-3	51,303,388	7,628,671,504	51,193,662	7,442,138,700 (97.55%)	7,108,738,685 (93.18%)	4,248,139,426 (55.69%)	48,728,948 (95.77%)	46,580,671 (91.54%)
TL-1	53,728,490	7,983,823,434	53,609,850	7,782,090,571 (97.47%)	7,429,812,850 (93.06%)	4,508,645,123 (56.47%)	48,895,354 (91.49%)	44,125,432 (82.56%)
TL-2	48,511,592	7,230,580,616	48,384,742	7,041,331,495 (97.38%)	6,713,834,168 (92.85%)	4,035,618,474 (55.81%)	45,033,230 (93.36%)	42,700,246 (88.53%)
TL-3	53,850,276	8,020,766,562	53,729,084	7,827,485,412 (97.59%)	7,484,806,819 (93.32%)	4,541,433,778 (56.62%)	49,703,779 (92.99%)	46,478,435 (86.96%)

### Identification of DEmiRNAs in the Lung

**Figure 3:**
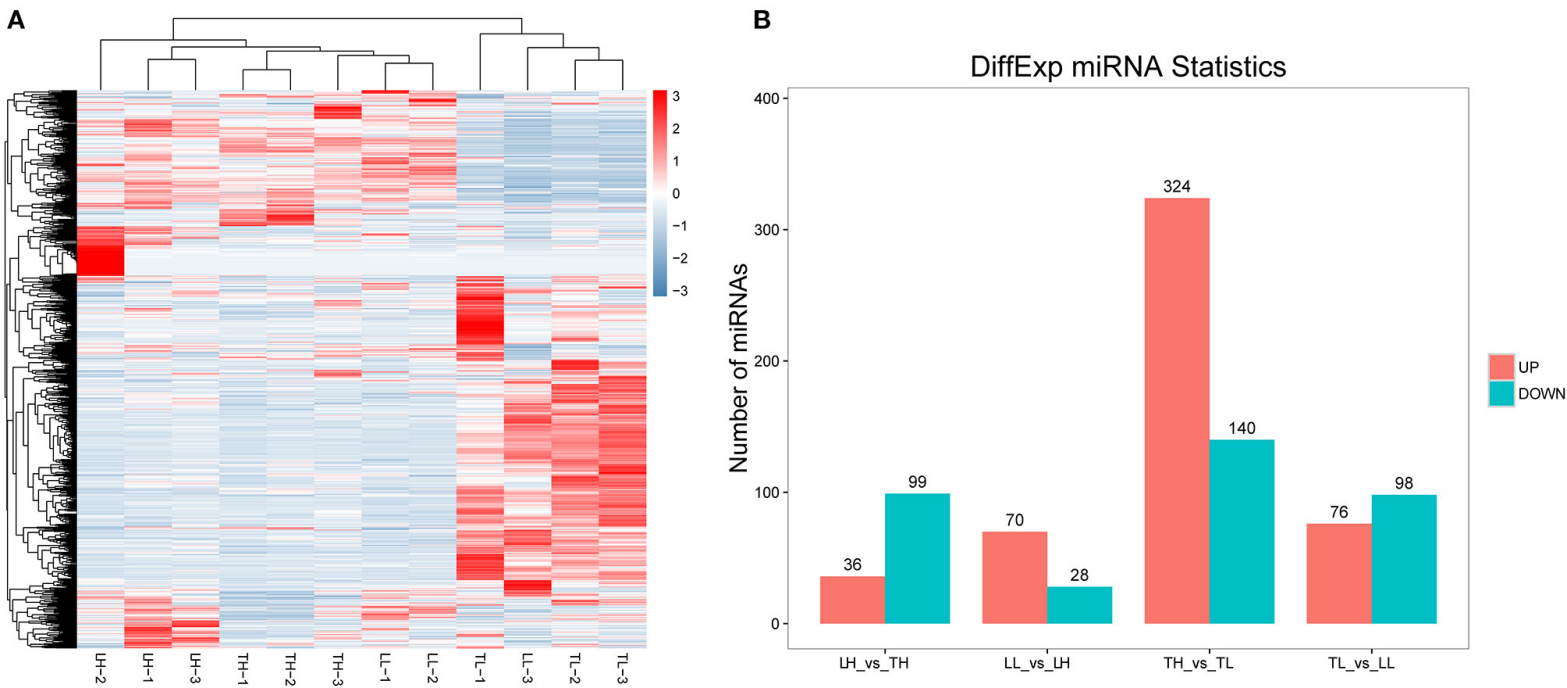
miRNA expression results among the four groups by RNA-seq. **(A)** The heatmap shows the relative expression patterns of DEmiRNAs among the four groups. Each column represents a sample, and each row represents the expression levels of a single miRNA in various samples. The color scale of the heat map ranges from blue (low expression) to red (high expression). **(B)** The histogram shows the number of DEmiRNAs identified among the four groups.

**Figure 4:**
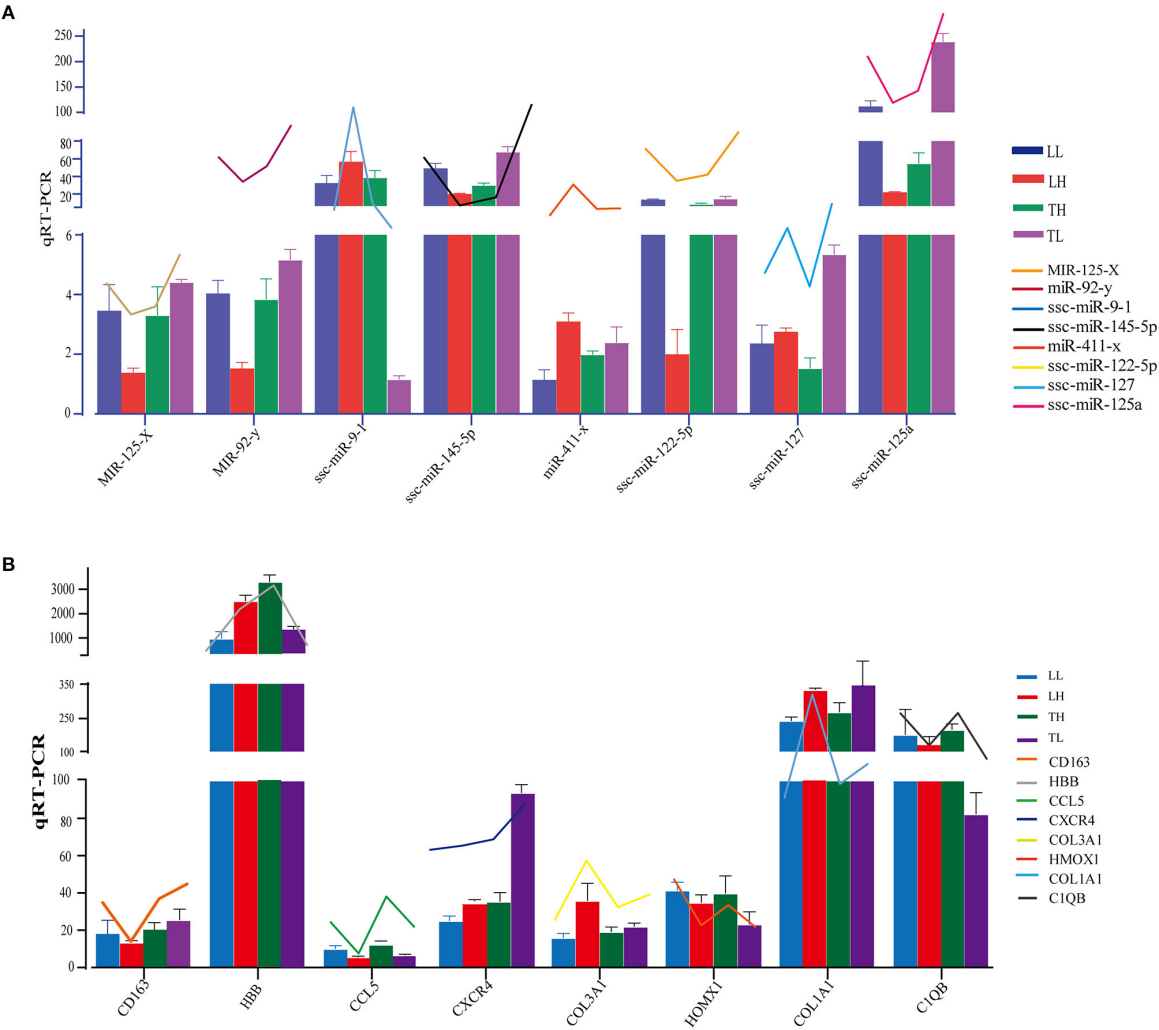
Expression patterns of randomly selected DEmRNAs and DEmiRNAs. **(A)** Eight mRNA expression levels were confirmed by qRT-PCR in comparison to corresponding data detected in mRNA-Seq. GAPDH was used as control. **(B)** Eight miRNA expression levels were confirmed by qRT-PCR in comparison to corresponding data detected in mRNA-Seq. U6 was used as control. The broken line indicates the change in transcript level according to the FPKM value of mRNA-seq and miRNA-seq. Three biological replicates with three technical replicates each were used. The values represent the mean ± SE (*n* = 3).

A total of 12 cDNA libraries were sequenced from lung tissues. In the miRNA-seq data, 10,810,538–14,920,316 clean reads were obtained by removing low-quality data and data with sequences shorter than 18 nt and longer than 30 nt, and 94.380–97.30% clean reads were obtained and mapped (Table 2). A total of 464 DEmiRNAs (324 up- and 140-downregulated) were identified in the TH group compared to the TL group (Figure 3, Supplementary Table 4 in Supplementary Materials 1, 4). Eight miRNAs were randomly selected and detected using qRT-PCR to validate the accuracy of the sequencing data. Our verification test indicated that the qRT-PCR results were consistent with the miRNA-seq data (Figure 4B).

**Table 2:** Overview of the reads and quality control of the 12 libraries of the miRNA sequencing from swine lung tissue.

**Sample**	**Clean_reads**	**High_quality**	**Smaller_than_18nt**	**Clean reads**	**Match**	**Ratio (%)**
LL-1	11,823,173 (100%)	11,800,040 (99.8043%)	117,090 (0.9923%)	147,719,777	122,350,552	82.83
LL-2	11,353,289 (100%)	11,339,875 (99.8818%)	90,118 (0.7947%)	11,422,900	9,303,536	81.45
LL-3	13,427,720 (100%)	13,404,468 (99.8268%)	107,640 (0.8030%)	10,937,366	9,065,662	82.89
LH-1	13,721,166 (100%)	13,702,350 (99.8629%)	303,894 (2.2178%)	13,042,101	10,696,421	82.01
LH-2	13,752,929 (100%)	13,569,709 (98.6678%)	261,550 (1.9275%)	13,042,192	11,012,185	84.44
LH-3	13,530,906 (100%)	13,507,736 (99.8288%)	153,784 (1.1385%)	12,902,983	10,703,895	82.96
TH-1	12,421,634 (100%)	12,401,306 (99.8364%)	165,479 (1.3344%)	13,093,151	10,784,226	82.37
TH-2	12,972,521 (100%)	12,954,856 (99.8638%)	247,892 (1.9135%)	11,909,246	9,793,972	82.24
TH-3	13,503,266 (100%)	13,476,246 (99.7999%)	161,856 (1.2010%)	12,330,439	10,255,345	83.17
TL-1	10,810,538 (100%)	10,788,450 (99.7957%)	132,232 (1.2257%)	12,998,511	10,664,603	82.04
TL-2	14,920,316 (100%)	14,892,778 (99.8154%)	388,770 (2.6105%)	10,283,765	8,634,859	83.97
TL-3	12,392,682 (100%)	12,373,187 (99.8427%)	226083 (1.8272%)	14,056,433	11,724,322	83.41

### Functional Analysis of DEmRNAs

**Figure 5:**
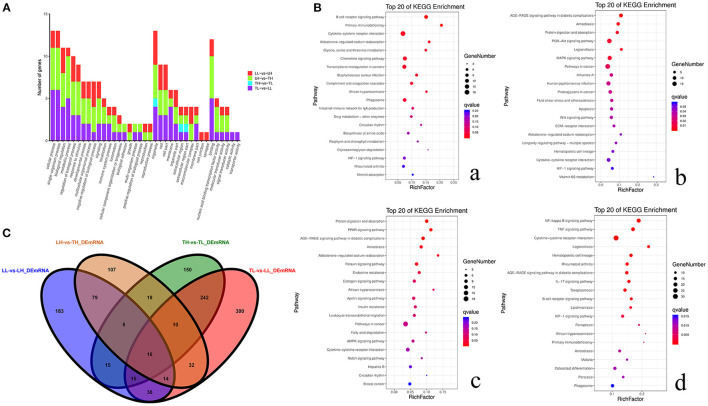
Functional annotation analysis of DEmRNAs in porcine lungs among the four groups. **(A)** Histogram of GO annotation results of DEmRNAs. The abscissa is the second level GO term, and the ordinate is the number of DEmRNAs in the term. Red indicates the number of DEmRNAs between Landrace pigs raised in lowlands (LL) and Landrace pigs raised in highlands (LH) groups, green indicates the number of DEmRNAs between LH and Tibetan pigs raised in highlands (TH) groups, blue indicates the number of DEmRNAs between TH and Tibetan pigs raised in lowlands (TL) groups, and purple indicates the number of DEmRNAs between TL and LL groups. **(B)** Top 20 KEGG enrichment pathways of DEmRNAs. The ordinate is the pathway, and the abscissa is the enrichment factor. Darker colors indicate smaller q-values. **a**. Pathway enrichment analysis of DEmRNAs between TH and TL. **b**. Pathway enrichment analysis of DEmRNAs between TH and LH. **c**. Pathway enrichment analysis of DEmRNAs between LH and LL. **d**. Pathway enrichment analysis of DEmRNAs between TL and LL. **(C)** Venn diagram of mRNA interactions based on the overlapping mRNAs among the four groups.

GO and KEGG enrichment analyses showed that most DEmRNAs were involved in cellular processes and pathways related to cytokine-cytokine receptor interaction, the PI3K-Akt signaling pathway, and pathways in cancer (Figure 5). Interestingly, a number of genes were mainly enriched in “response to stimulus (GO: 0050896)” of biological process among the four groups. GO: 0001071 is associated with nucleic acid binding transcription factor activity and was significantly enriched between the TH and LH groups. The top 20 pathways with the most significant enrichment were obtained. KEGG enrichment results revealed that most of these genes were significantly enriched in cancer pathways among Landrace pigs (LH and LL) (breast cancer and transcriptional misregulation in cancers) or high-altitude groups (LH and TH) (proteoglycans in cancer, pathways in cancer, breast cancer). A number of genes were significantly enriched in cytokine–cytokine receptor interaction, hematopoietic cell lineage, and African trypanosomiasis among Tibetan pigs and Landrace pigs in the high- or low-altitude groups. Six pathways were significantly enriched among the high- (TH and LH) or low-altitude (TL and LL) groups, and 15 pathways were significantly enriched between the Tibetan pig (TH and TL) and Landrace pig (LH and LL) groups.

### Identification and Prediction Targets of DEmiRNAs

**Figure 6:**
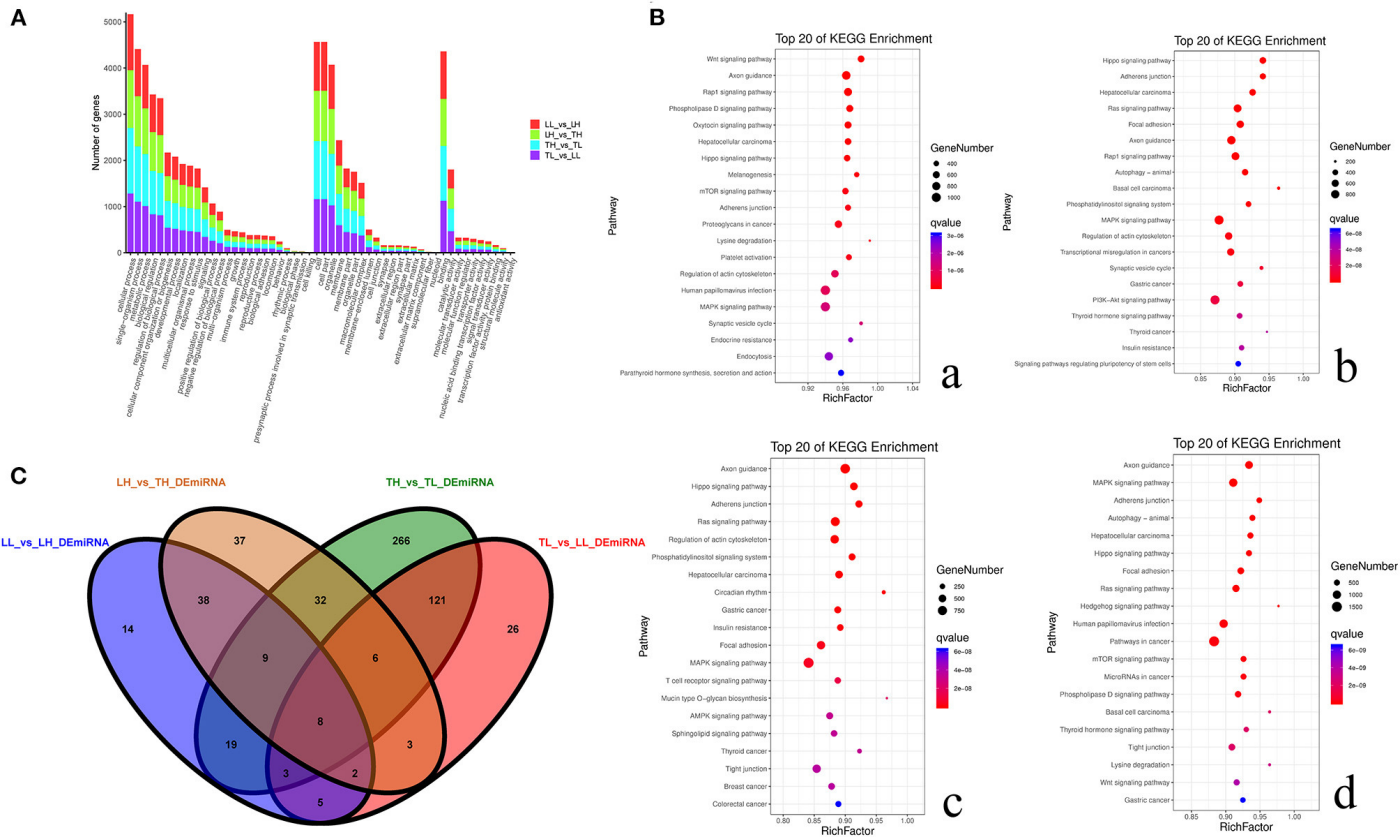
Functional annotation analysis of DEmiRNA-target genes in porcine lungs among the four groups. **(A)** Histogram of GO annotation results of DEmiRNA-target genes. The abscissa is the second-level GO term, and the ordinate is the number of DEmiRNA-target genes in the term. Red indicates the number of DEmiRNA-target genes between Landrace pigs raised in lowlands (LL) and Landrace pigs raised in highland (LH) groups, green indicates the number of DEmiRNA-target genes between LH and Tibetan pigs raised in highland (TH) groups, blue indicates the number of DEmiRNA-target genes between TH and Tibetan pigs raised in lowland (TL) groups, and purple indicates the number of DEmiRNA-target genes between TL and LL groups. **(B)** Top 20 KEGG enrichment pathways of DEmiRNA-target genes. The ordinate is the pathway, and the abscissa is the enrichment factor. Darker colors indicate smaller q-values. **a**. Pathway enrichment analysis of DEmiRNA-target genes between TH and TL. **b**. Pathway enrichment analysis of DEmiRNA-target genes between TH and LH. **c**. Pathway enrichment analysis of DEmiRNA-target genes between LH and LL. **d**. Pathway enrichment analysis of DEmiRNA-target genes between TL and LL. **(C)** Venn diagram of miRNA interactions based on the overlapping miRNAs among the four groups.

A total of 59,636 target DEmRNAs of 1,630 DEmiRNAs (365 functionally annotated miRNAs, 989 known miRNAs and 276 novel miRNAs) were analyzed (Supplementary Materials 5). In addition, multiple pathways and GO terms were associated with hypoxia traits. The analysis revealed KEGG pathways that were significantly related to genes targeted by DEmiRNAs, and the Wnt signaling pathway, metabolic pathway and hepatocellular carcinoma were the most significantly related (Figure 6). Interestingly, the results showed that the targets were primarily enriched in terms related to hypoxia adaptation. ssc-miR-210, ssc-miR-101, ssc-miR-7136-5p, ssc-miR-10b, ssc-miR-206, ssc-miR-1343, ssc-miR-142-5p, ssc-miR-421-5p, and ssc-miR-4331 were identified as key miRNAs. Functional assessment showed that 100, 56, and 104 putative targets were mainly enriched in the HIF, PI3K-Akt, and MAPK signaling pathways, respectively.

### Screening of Differentially Expressed Hypoxia-Related mRNA Target miRNAs and Their Functional Enrichment Analysis

**Figure 7:**
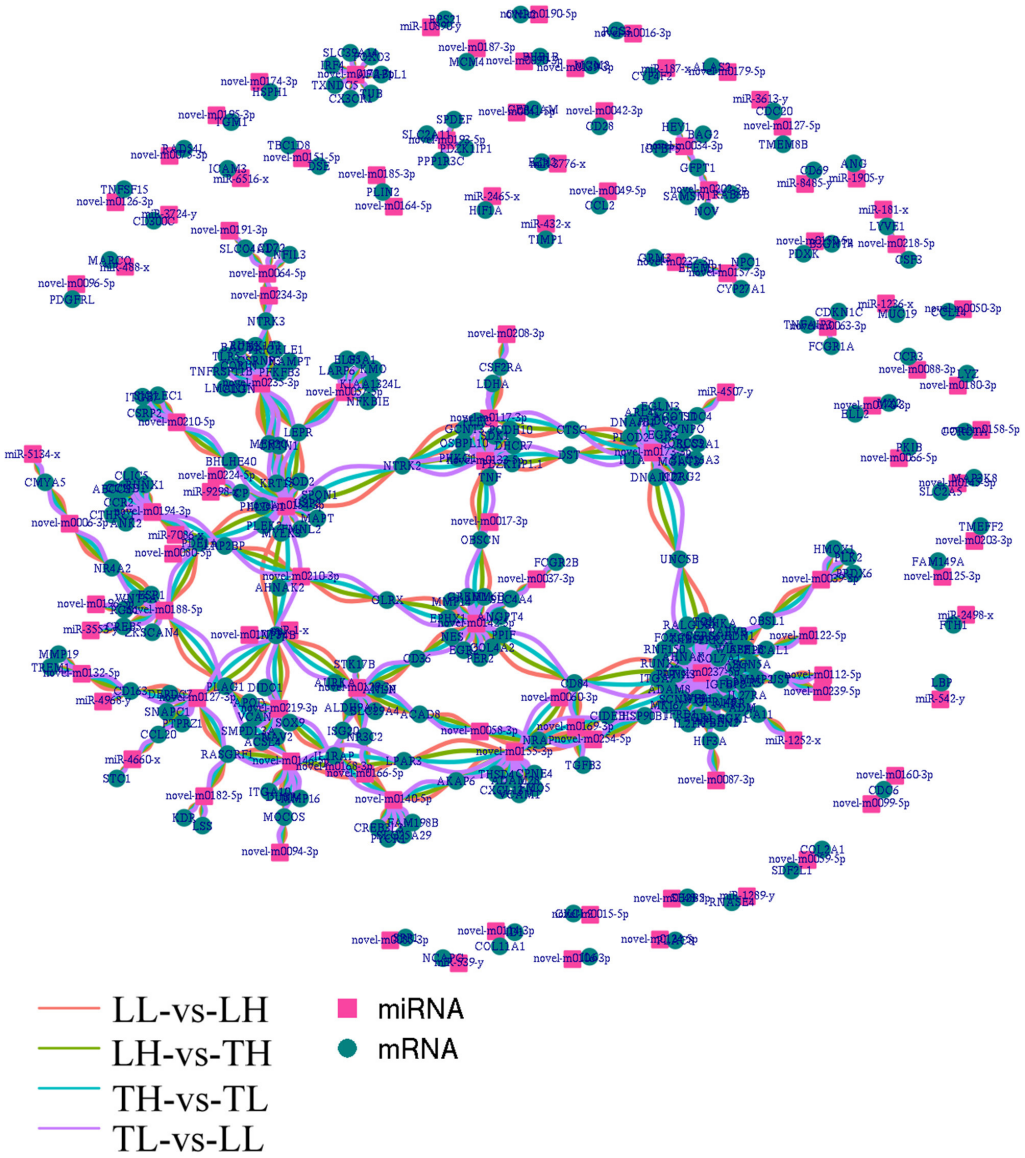
Integrated miRNA-target negative correlation regulatory network. The square nodes represent miRNAs, and the circle nodes represent target genes. The differentially expressed miRNAs/target genes are highlighted as follows: red indicated differential expression between Landrace pigs raised in lowlands (LL) and Landrace pigs raised in highland (LH), green indicates differential expression between LH and Tibetan pigs raised in highland (TH), blue indicates differential expression between TH and Tibetan pigs raised in lowland (TL), and purple indicates differential expression between TL and LL.

Functional analysis was conducted to understand the pathways and molecular interactions of DEmRNAs and DEmiRNAs. The DEmRNAs were enriched in a number of important pathways related to hypoxia, and we identified 273 significant DEmRNAs involved in hypoxia adaptation among the four groups (Supplementary Material 6). We predicted potential target miRNAs of mRNAs according to the negative regulatory effects of miRNAs on mRNAs, which were further considered veritable miRNA–mRNA pairs. To further reveal the regulatory relationship of node mRNAs and non-coding miRNAs, the resulting potential regulatory networks of miRNA-target genes associated with hypoxia-genes were constructed (Figure 7). The target DEmRNAs of DEmiRNAs were assessed using KEGG and GO analyses. The results indicated that 71.09, 17.00, and 11.90% (total of 273) of the genes were enriched in the biological process (BP), cell component (CC), and molecular function (MF) categories, respectively, in the TH-vs.-TL comparison (*p* < 0.05). A number of genes were targeted by hub miRNAs, such as novel-m0237-5p, novel-m0173-3p, and novel-m0142-5p, which had 45, 19, and 14 target mRNAs among the four groups, respectively. Furthermore, miR-2465-x targeted HIF-1α, while novel-m0087-3p and novel-m0237-5p targeted *HIF*-3α.

### Construction of the Coexpression Network Between DEmRNAs and DEmiRNAs in Response to Hypoxia

**Figure 8:**
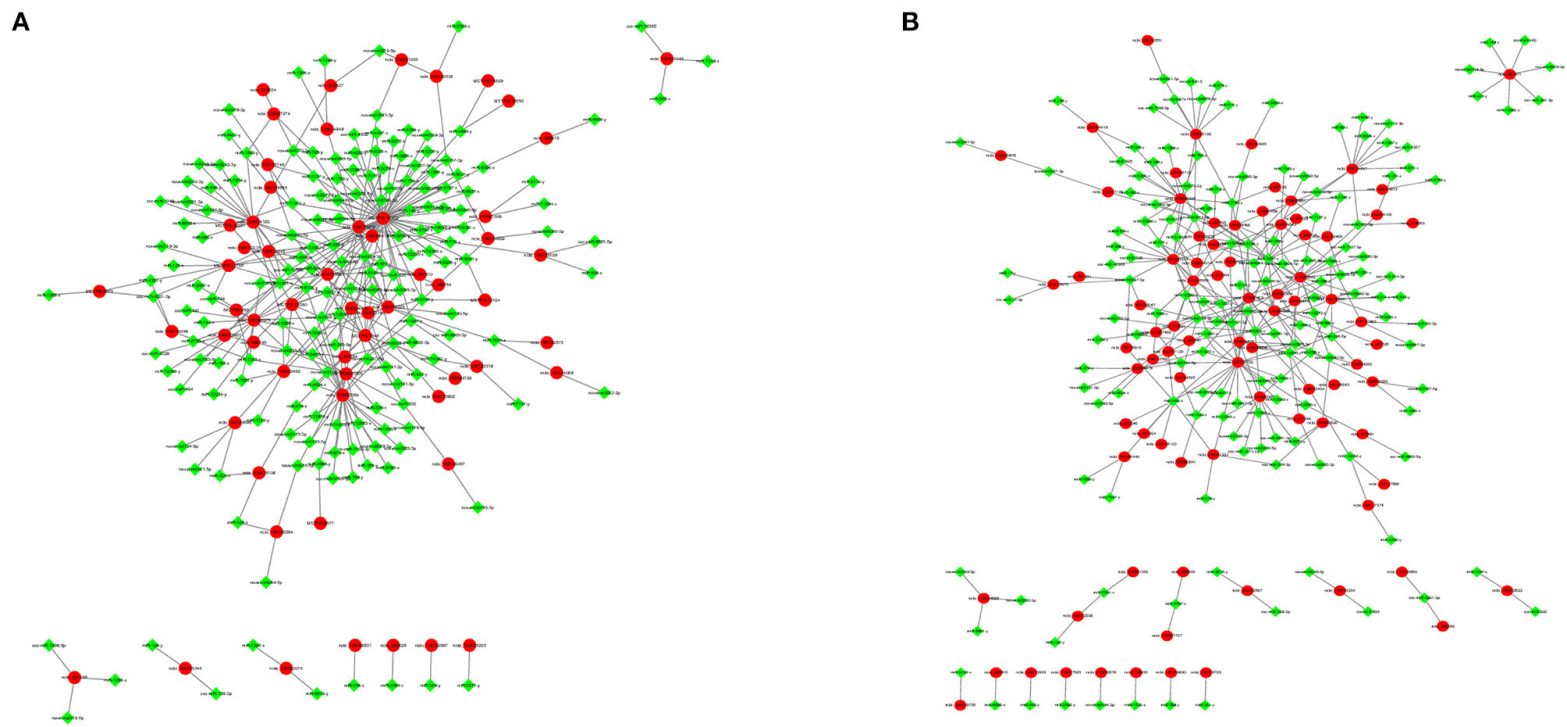
Gene coexpression network analyses. Red nodes indicate hypoxic DEmRNAs, and green nodes indicate DEmiRNAs. **(A)** Gene coexpression network analyses of DEmRNAs and DEmiRNAs. **(B)** Gene coexpression network analyses of hypoxic DEmRNAs and DEmiRNAs.

To explore the relationship between miRNAs and mRNAs in a hypoxic environment, a coexpression network of DEmRNAs and DEmiRNAs was constructed, and the top 300 relationship pair network diagrams are listed (Figure 8A, Supplementary Material 7.1). The intersection of differentially expressed hypoxia mRNAs and miRNAs identified from the four group comparisons represented their differential expression in pig lungs with increasing altitude. *TAR1*-*A, GPD*1, *ST8SIA*5, and *LENG*8 were selected as the most affected mRNAs, and there were strong correlations with a number of miRNAs. Furthermore, a coexpression network of 273 hypoxic DEmRNAs and DEmiRNAs was constructed (Figure 8B, Supplementary Material 7.2). *MEF*2*C*, AKAP6, *NTRK*2, *MAPT*, and *GPR*146 were selected as the most affected mRNAs, and there were strong correlations with a number of miRNAs.

## Discussion

A high-altitude environment plays an important role in the adaptation of native species, and it may modify gene transcription and may irreversibly affect specific phenotypes (Zhang et al., 2015; Ni et al., 2019). We used a complete migrant design to evaluate genes interacting with the environment and selected Tibetan pigs and Landrace pigs in both their native altitude environments and as migrants in a non-native environment. Our previous research identified that Tibetan pigs have heavier and wider lungs, thicker alveolar septa, and a denser vascular network than Landrace pigs. The hemoglobin (HGB) and mean corpuscular hemoglobin concentration (MCHC) of high-altitude pigs (Tibetan and Landrace pigs) were significantly higher than those of low-altitude pigs (Yang et al., 2021). We next investigated whether there are gene expression changes specific to Tibetan pigs that are responsible for hypoxic adaptation. Sequencing of multiple pigs from different breeds revealed that certain genomic regions, including genes involved in the hypoxia response, were under selection in Tibetan pigs (Zhang B. et al., 2016; Zhang et al., 2017, 2019a). We screened for key genes related to hypoxic adaptation through genotype and environment interaction effects *via* RNA-seq analyses. Several pathways were enriched in DEmRNAs among Tibetan pigs and Landrace pigs at different altitudes, including the VEGF signaling pathway, PI3K-AKT signaling pathway, and mTOR signaling pathway (Ai et al., 2015; Zhang et al., 2017). Moreover, GO enrichment analysis revealed that these DEmRNAs were associated with vascular regulation, regulatory region DNA binding, or extracellular region. The identified hypoxia-related signaling pathways may form a complex cascade of responses that occur in hypoxic conditions in Tibetan pigs to reduce the risk of pulmonary damage.

Hypoxia-regulated miRNAs play vital roles in cell survival and have been implicated in the regulation of both upstream and downstream HIF-1 signaling pathways under hypoxic conditions. For example, miR-199a, miR-17-92, and miR-20b induce HIFs (Dai et al., 2015; Chen et al., 2016; Danza et al., 2016). *HIF*-1 regulates the expression of various genes to protect cells from hypoxic injury through cell apoptosis, glucose metabolism, and mitochondrial function (Bhattarai et al., 2018; Yu et al., 2018, 2020). *HIF*-1α is a potential therapeutic proangiogenic molecule that regulates the levels of *VEGF* to elevate interstitial pressure (Zhi et al., 2018; Lin et al., 2019). Several putative target genes (*FOXO*3, *RASGRF*1, and *CX3CR*1) that are regulated by ssc-miR-214, ssc-miR-320, and ssc-miR-101 have been found to be involved in the HIF-1 related signaling pathway. miR-210 is located on human chromosome 11p15.5 and correlates with angiogenesis and *VEGF* regulation in breast cancer patients (Forkens et al., 2008; Dai et al., 2015; Tang et al., 2018; Zhang H. et al., 2019). In the present study, the expression of miR-210-x and miR-210-z was significantly lower in TH than in TL but not significantly different in the other groups, which may play vital roles in the expression of proteins in homology-dependent repair pathways and nucleotide excision repair pathways to reverse cellular DNA damage in the lungs of Tibetan pigs during hypoxia (Crosby et al., 2009; Hui et al., 2019). *HBB* is involved in the malaria reference pathway and downregulates *IL*-6, which is a key gene in the HIF-1 pathway. Comparison of *HBB* expression between Tibetan pigs and Landrace pigs showed that among the beta globin amino acid substitutions at positions 58, 75, 119 and 137, the replacement of alanine at position 137 with valine and the locus mutation improved the affinity of HGB and O_2_ (Zhang B. et al., 2016). The expression of the HBB gene in Tibetan pigs (TH and TL) was significantly higher than that in Landrace pigs (LH and LL), agreeing with a similar trend previously reported by other authors, and there was similar variation in the HGB concentration in Tibetan pigs (Taliercio et al., 2013; Zhang B. et al., 2016; Yang et al., 2021), indicating that hypoxia transcriptionally upregulates HBB to increase HGB in the blood to ensure the transport of blood and nutrients. These findings may (Jang et al., 2014; Zhang G. et al., 2016; Cai et al., 2018) explain why Tibetan pigs have better adaption than Landrace pigs in hypoxic environments regardless of altitude.

The PI3K/Akt pathway is an intracellular signaling pathway that is promoted by several biological molecules, including calmodulin, insulin-like growth factor (IGF), and multiple EGF-like domains 6 (MEGF6) (Pompura and Dominguez-Villar, 2018; Ellis and Ma, 2019; Revathidevi and Munirajan, 2019; Zhang et al., 2020). *IGF*2 is the target gene of miR-506-y and ssc-miR-181d-3p. The expression levels of *IGF*2 and *MEGF*6 were significantly upregulated in LH compared to LL, but no differences were found in the Tibetan pigs (TH and TL). We hypothesized that these genes may induce the growth, proliferation, and differentiation of tumor cells in the lungs of Landrace pigs living in a hypoxic environment (Mohlin et al., 2013). Activated Akt induces various biological processes, including activating mTOR, localizing FOXO to the cytoplasm, and activating cAMP-response element binding protein (CREB) (Zhang et al., 2011; Gaecía-Morales et al., 2017; Marquard and Jücker, 2020). The FOXO signaling pathway was also enriched in a comparison of pigs living at different altitudes. It has been shown that alcohol suppresses P450 oxidoreductase (POR) and glutathione reductase (GSR) gene expression by upregulating miR-214, which induces oxidative stress and plays a crucial role in adaptation to hypoxia (Zhou et al., 2013; Dong et al., 2014; Stefanetti et al., 2018; Li et al., 2019). *FOXO*3 is a targeted gene of ssc-miR-214-3p, and the expression of *FOXO*3 in the TH group was significantly higher than that in the TL group, but not significantly different between the LH and LL groups. Changes in ssc-miR-214-3p expression may inhibit the cell cycle and promote apoptosis, thereby inhibiting cell proliferation through *FOXO*3 prolyl hydroxylation in hypoxic conditions. The regulation of the expression levels of *IGF*2, *MEGF*6, and *FOXO*3 through miRNAs may lead to the better adaption of Tibetan pigs in hypoxic environments compared to the Landrace breed.

Collagens, such as *COL1A*1, *COL1A*2, and *COL*3*A*1, are widely represented in ECM–receptor interactions and focal adhesion pathways (Gelse et al., 2003), and their expression was significantly higher in the LH group than in the LL group but not significantly different between the TH and TL groups. All of these genes function as mechanoreceptors and may provide a force-transmitting physical link between the EMC and cytoskeleton, indicating that enhanced expression of *COL1A*1, *COL1A*2, and *COL*3*A*1 may be another reason for the superior adaption to hypoxic conditions of TH. Our study revealed that high expression of fibroblast growth factors (such as *FGF*1, *FGF*2, *FGF*9) was higher in the native groups (TH and LL) than in the migrated groups (TL and LH), which was alleviated by activating *AKT*3 (Pompura and Dominguez-Villar, 2018; Revathidevi and Munirajan, 2019). These findings indicated that Tibetan pigs may increase the expression of *FGF*1 and the cross-sectional area of a blood vessel to increase blood flow in response to hypoxia (Karar and Maity, 2011; Kir et al., 2018; Sajib et al., 2018).

*GPR*146 may be upregulated by a number of miRNAs (such as miR-8903, miR-11972 and miR-466-x) under hypoxic stimulation and has been suggested to be an important hypoxia-inducible gene in recent years. C-peptide inhibits low O_2_-induced ATP release in erythrocytes as a putative ligand of *GPR*146, which was consistent with our results (Richards et al., 2014). Ncbi_397391 (*MMP*2), ncbi_102159047 (*FOXC*1), ncbi_100738910 (*PRRX*1), and ncbi_100520318 (*TUB*) are potentially regulated by novel-m0237-5p. In the present study, *MMP*2 expression was significantly higher in the LH group than in the LL group, but no significant differences were found between the TH and TL groups. The expression of *MMP*2 showed a similar tendency to the results of alveolar septum thickness among the four groups, indicating that *MMP*2 activities may lead to the widening of the alveolar wall and septum as well as alveolar structure damage and collapse of the alveolar space with remarkable fibrosis in Landrace pigs (Tan et al., 2006).

## Conclusion

The comparisons between Tibetan pigs and Landrace pigs from high or low altitudes revealed genes and regulatory pathways with possible adaptive changes in response to high-altitude hypoxia. We identified several molecular pathways and hypoxia genes showing adaptive changes in the lung, including increased blood circulation and regulation of blood pressure and circulation as well as regulation of HGB concentration and angiogenesis. Integrated analysis of mRNAs and miRNAs demonstrated that a number of hypoxia genes may be regulated by miRNAs and participate in the hypoxic regulation of the lung. For example, novel-m0237-5p may potentially upregulate the expression levels of *MMP*2, resulting in widened alveolar septum and alveolar structure damage. These results provide a better understanding of the molecular mechanisms regulating the hypoxia response in the lungs of Tibetan pigs and will help to prevent damage to the lungs caused by hypoxia.

## Data Availability Statement

The datasets presented in this study can be found in online repositories. The names of the repository/repositories and accession number(s) can be found in the article/Supplementary Material.

## Ethics Statement

The animal study was reviewed and approved by Ministry of Science and Technology of the People's Republic of China (Approval number: 2006-398). Written informed consent was obtained from the owners for the participation of their animals in this study.

## Author Contributions

SZ was the overall project leader who provided financial support and experimental conception. YY was involved in data analyses, statistical analyses, language revisions, journal selection, and manuscript submissions and revisions. HY, TJ, and TY contributed to the experimental design and implementation. CG contributed to the supervision and assistance of students in managing animals and collecting and analyzing samples. YL was responsible for the trial implementation, supervision of students collecting and analyzing samples, and manuscript preparation. YC contributed to supervision of sample collection and analysis and manuscript editing. All authors contributed to the article and approved the submitted version.

## Funding

This study was supported by the National Natural Science Foundation of China (31760644, 32060730).

## Conflict of Interest

The authors declare that the research was conducted in the absence of any commercial or financial relationships that could be construed as a potential conflict of interest.

## Publisher's Note

All claims expressed in this article are solely those of the authors and do not necessarily represent those of their affiliated organizations, or those of the publisher, the editors and the reviewers. Any product that may be evaluated in this article, or claim that may be made by its manufacturer, is not guaranteed or endorsed by the publisher.

## Abbreviations

TH: Tibetan male piglets from the highlands
LL: Landrace male piglets from the lowlands
TL: Tibetan male piglets migrated to the lowlands
LH: Landrace male piglets migrated to the highlands.
