# Supplementary material for: The Expression Regulatory Network in the Lung Tissue of Tibetan Pigs Provides Insight Into Hypoxia-Sensitive Pathways in High-Altitude Hypoxia
Source: Front Genet. 2021 Oct 7;12:691592. doi: 10.3389/fgene.2021.691592 (PMC8529057; doi:10.3389/fgene.2021.691592)
Supplement: Supplementary file 1 [file Data_Sheet_1.docx]

Supplementary Table 1 Primers used to detect mRNAs from the lungs of pigs by qRT-PCR

| Genes | Primer sequences (5'-3') | Annealing temperature/℃ |
| --- | --- | --- |
| *Hmox1* | F: CAACCCTGTGAATGCAACCG | 60 |
|  | R: CACATGCCCAACAAGGAAGC |  |
| *COL3A1* | F: GGTTTGAAGATGAGGAGCTACAGCA | 60 |
|  | R: CCAGTCCCAGACCCTGTCAAAGAT |  |
| *COL1A1* | F: GGGCAACAGCAGATTCACCTAC | 60 |
|  | R: GGTCAGC A T CACCGATGTCCAA |  |
| *CD163* | F: ATTCATCATCCTCGGACCCAT | 60 |
|  | R: CCCAGCACAACGACCACCT |  |
| *CCL5* | F: CAC ACC CTG CTG TTT TTCC | 60 |
|  | R: CCA TTTCTT CTC TGG GTT GG |  |
| *SLPI* | F: CAAGTGCACAAGTGACTGGC | 60 |
|  | R: GGCCATAGACCACTGGACAC |  |
| *HBB* | F: CTCCTGGGCAACGTGATAGT | 60 |
|  | R: GGTCAGAGGAAAAAGGGCTCCTCCT |  |
| *CXCR4* | F: ATCAGAAGCGCAAAGCTCTC  R: GAGGATGAAGGAGTCGATGC | 60 |
| *C1QB* | F: ACTTCCGCTTTGGACTGAGAG  R: GCTGCTTCCTGGGAACCTGAT | 60 |
| *IFI6* | F: AAGGCGGTATCGCTCTTCTT  R: TTCTGTTTTGCTTGGTTTTGTTT | 60 |
| *AKR1CL1* | F: AACTTCGGCTGGATTATGTTGA  R: AAAATAACTTTCCCACTGGCATC | 60 |
| *β-actin* | F: CAGTCGGTTGGATGGAGCAT  R: AGGCAGGGACTTCCTGTAAC | 60 |

Supplementary Table 2 Primers used to detect miRNAs from the lungs of pigs by qRT-PCR

| Genes | Primer sequences |
| --- | --- |
| Ssc-miR-122-5P | TGGAGTGTGACAATGGTGTTT |
| miR-92-y | TATTGCACTTGTCCCGGCCT |
| Ssc-miR-127 | TCGGATCCGTCTGAGCTTG |
| miR-125-x | TCCCTGAGACCCTAACTTGTG |
| miR-411-x | ATAGTAGACCGTATAGCGTACG |
| ssc-miR-125a | TCCCTGAGACCCTTTAACCT |
| ssc-miR-145-5p | GTCCAGTTTTCCCAGGAATCC |
| ssc-miR-9-1 | GCTCTTTGGTTATCTAGCTGTA |
| ssc-miR-455-3p | GCAGTCCATGGGCATATACAC |
| miR-451-x | GCAAACCGTTACCATTACTGAG |
| U6 | F: GGAACGATACAGAGAAGATTAGC  R: TGGAACGCTTCACGAATTTGCG |

Supplementary Table 3 The number of DEmRNAs were identified.

| **Comparison group** | **DEmRNAs** | |
| --- | --- | --- |
|  | **Upregulated** | **Downregulated** |
| LYD-vs-TGN | 114 | 167 |
| LJC-vs-LYD | 258 | 105 |
| TGN-vs-TJC | 247 | 224 |
| TJC-vs-LJC | 257 | 408 |

Supplementary Table 4 The number of DEmiRNAs were identified.

| **Comparison group** | **DEmiRNAs** | |
| --- | --- | --- |
|  | **Upregulated** | **Downregulated** |
| LYD-vs-TGN | 36 | 99 |
| LJC-vs-LYD | 70 | 28 |
| TGN-vs-TJC | 324 | 140 |
| TJC-vs-LJC | 76 | 98 |
