# Supplementary material for: The Expression Regulatory Network in the Lung Tissue of Tibetan Pigs Provides Insight Into Hypoxia-Sensitive Pathways in High-Altitude Hypoxia
Source: Front Genet. 2021 Oct 7;12:691592. doi: 10.3389/fgene.2021.691592 (PMC8529057; doi:10.3389/fgene.2021.691592)

Supplementary Figure 1. Density plot of mRNA interactions based on the overlapping mRNAs among the four groups.


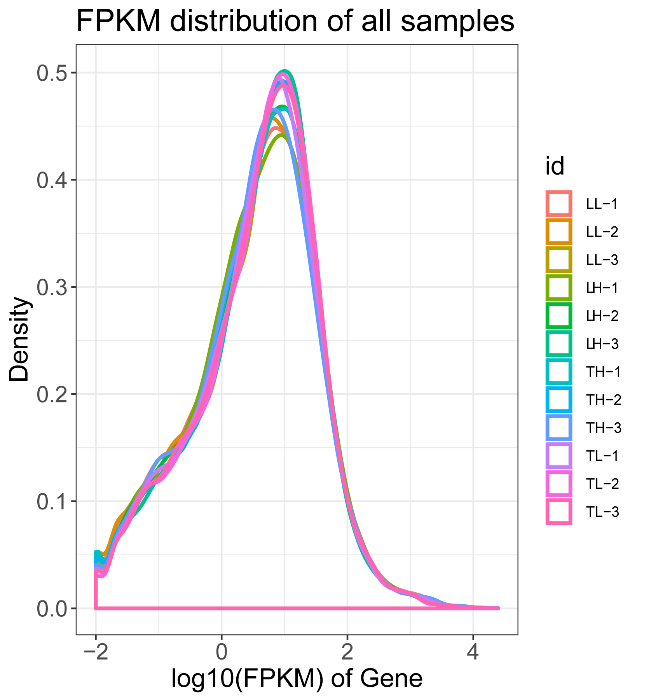


Supplementary Figure 2. Comparison of the overall expression levels among four groups.


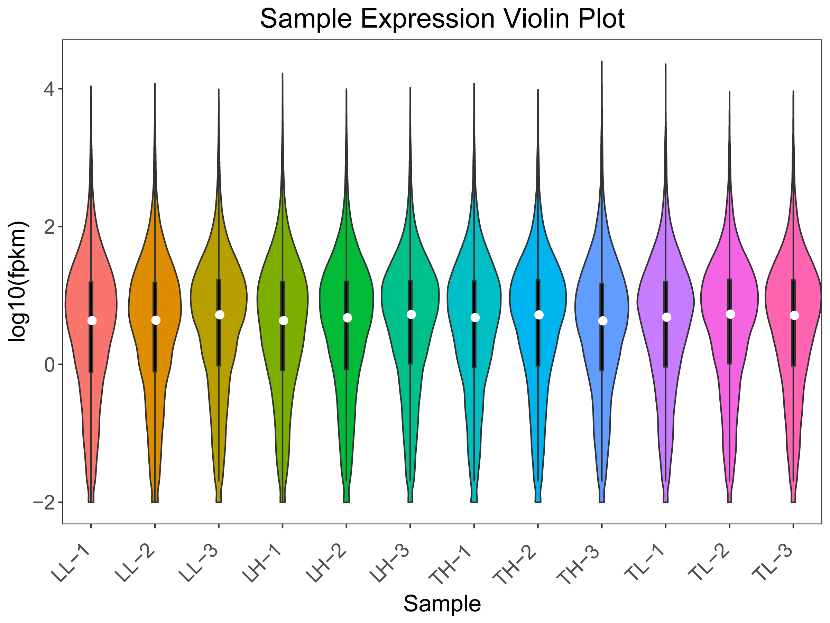


Supplementary Figure 3. Differential expression of mRNAs among four groups. P-values and log2FC values were used to screen for differentially expressed transcripts according to the following thresholds: P < 0.05 and |log2FC| > 1.


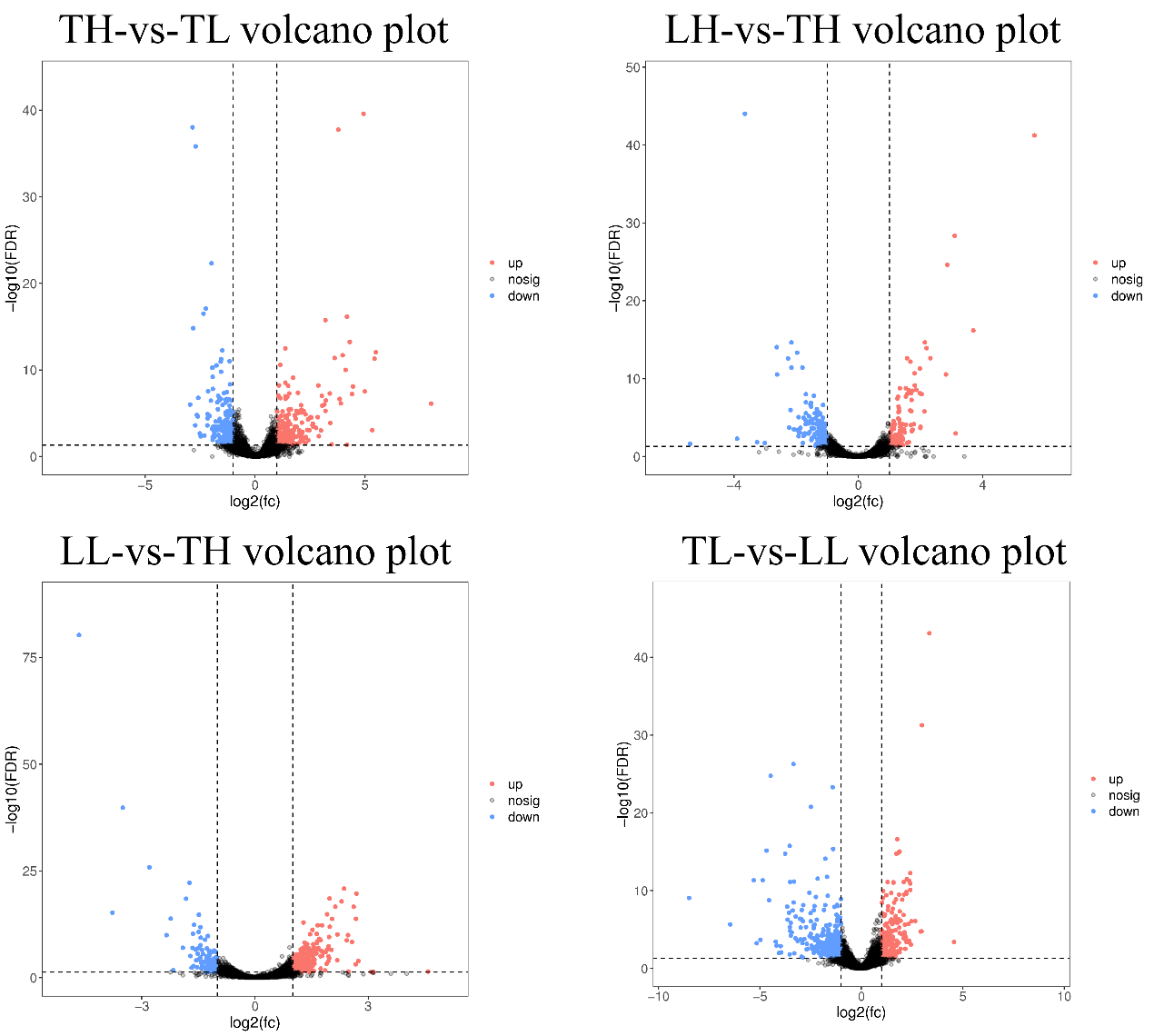

Supplement: Supplementary file 2 [file Data_Sheet_2.docx]
